# Fabrication of Cu_2_ZnSnS_4_ Light Absorber Using a Cost-Effective Mechanochemical Method for Photovoltaic Applications

**DOI:** 10.3390/ma15051708

**Published:** 2022-02-24

**Authors:** Meenakshi Sahu, Vasudeva Reddy Minnam Reddy, Bomyung Kim, Bharati Patro, Chinho Park, Woo Kyoung Kim, Pratibha Sharma

**Affiliations:** 1 Department of Energy Science and Engineering, Indian Institute of Technology Bombay Powai, Mumbai 400076, India; meenakshisahu.chem@gmail.com; 2 Korea Institute of Energy Technology (KENTECH), 200 Hyukshin-ro, Naju 58330, Korea; 3 School of Chemical Engineering, Yeungnam University, Gyeongsan 38541, Korea; drmvasudr9@gmail.com (V.R.M.R.); billionp10@ynu.ac.kr (B.K.); 4 Centre for Research in Nanotechnology and Sciences Indian Institute of Technology Bombay Powai, Mumbai 400076, India; bharati@iitb.ac.in

**Keywords:** Cu_2_ZnSnS_4_, mechanochemical method, annealing, photovoltaic application

## Abstract

In the present study, we adopt an easy and cost-effective route for preparing Cu_2_ZnSnS_4_ (CZTS)-absorber nanoparticles by a mechanochemical method using non-toxic and environmentally benign solvents (butanol, methyl ethyl ketone, and ethanol). The gram-scale synthesis of absorber nanoparticles was achieved in a non-hazardous, zero-waste process without using high-vacuum equipment. The effects of annealing and Na incorporation on the properties of spin-coated CZTS thin films were scrutinized. The deposited samples showed kesterite crystal structure and single phase. The morphological results revealed an improvement in the surface morphology after annealing. The optical bandgaps of the thin films lied in the range of 1.50–1.57 eV with p-type nature. Finally, photovoltaic devices were fabricated, and their cell performance parameters were studied. An efficiency of 0.16% was observed. The present study provides a potential route for the cost-effective fabrication of CZTS-based photovoltaic devices.

## 1. Introduction

Kesterite-based Cu_2_ZnSnS_4_ (CZTS) materials have been extensively studied as substitutes to CdTe and Cu(In, Ga)Se_2_ (CIGS) compounds for thin-film-based solar cell applications. Owing to its remarkable properties such as earth-abundant and non-toxic elements, optimal optical bandgap (1.0–1.5 eV), excellent absorption coefficients (>10^4^ cm^−1^) [1,2], and desirable electrical properties, the CZTS has become an ideal absorber for thin-film-based photovoltaic applications. Currently, various fabrication techniques are being used to deposit kesterite thin films, including atom beam sputtering [3], hybrid sputtering [4], radiofrequency magnetron sputtering [5], co-evaporation [6], electron-beam-evaporated [7], electrodeposition [8], sol-gel [9], spray pyrolysis [10], and molecular ink techniques [11]. These processes involve either costly vacuum equipment or toxic, hazardous, and explosive solvents. Therefore, an environmentally friendly and non-hazardous approach for depositing kesterite thin films is of utmost importance.

Slurry or nanoparticle inks composed of solid particles, which provide an alternative for fabricating thin films for solar-cell applications, can be synthesized by the mechanochemical or ball-milling method [12]. The mechanochemical solid-state method is a direct reaction approach that solves major concerns associated with other non-vacuum techniques. It also offers many benefits such as cost-effectiveness, simplicity, low temperature, gram-scale synthesis, grinding of various powder mixtures (either compounds or elements), and making fine powders [13]. Several compounds, such as Cu(In, Ga)Se_2_ [14], CuInSe_2_ [15], and CdTe [16], have been synthesized by ball milling, and they have recently been employed to fabricate kesterite absorber thin films and kesterite-based photovoltaic devices. In 2011, Wang and Gong first reported that CZTS could be synthesized by planetary ball milling using its elemental precursors for 25 h [17]. In 2012, Gao et al. reported the preparation of high-purity Cu_2_ZnSn(S_x_, Se_1−x_)_4_ (CZTSSe) powders by milling for a short period (20 min) and then post-heating for 5 h at 550 °C under nitrogen [18]. Several researchers have reported the fabrication of photovoltaic devices from kesterite nanoparticle inks. Moon et al. reported a CZTS photovoltaic device with 5.14% efficiency using an absorber fabricated with ethanol-based nanoparticle ink by spin coating [19]. Further development with 7.1% efficiency was achieved by employing bandgap-graded CZTSSe absorber layers [20].

In addition to the selection of low-cost syntheses, alkali-metal-incorporation strategies have been used to increase the performance of photovoltaic cells. The use of alkali metals, particularly Na, has been observed to have positive effects on kesterite-based absorber layers and photovoltaic devices [21,22,23]. Experimental and theoretical studies have demonstrated that one of the limitations of the photoconversion efficiency of CZTS thin film-based devices is the existence of inherent defects at the interface and grain boundary, which can be effectively passivated by the incorporated Na. As a result, grain growth and (112) texturization improvement, scattering, and recombination centers can be reduced in the absorber layer, suppressing non-radiative recombination, reducing charge traps, and improving the electrical properties of the absorber layer [22,24,25,26,27]. Oo et al. reported grain growth in CZTS films prepared on soda-lime glass (SLG) or immersed in an aqueous solution of Na_2_S [28]. Furthermore, Zhou et al. described an efficient defect passivation procedure using CZTS:Na nanocrystals. The CZTS:Na-nanocrystal-based photovoltaic device demonstrated a 50% increase in device performance (~6%) compared to a non-passive CZTS-nanocrystal-based device (~4%) [29]. Sun et al. achieved a CZTS device efficiency of 6.2% using Na-doped Mo as the back-contact electrode on a flexible stainless-steel substrate [30]. Recently, Hao et al. reported an improved CZTS device efficiency from 2.37% to 3.26% [22]. Jiang et al. reported 2.3% CZTS device efficiency using a bifacial Na layer strategy [23]. These promising results indicate that the ball-milling process with the incorporation of Na may be a potential synthetic approach for the fabrication of kesterite-based photovoltaic absorbers.

In this study, CZTS kesterite material was prepared from a nano-powder ink-based approach with nonionic surfactants, low molecular weight binders, azeotropic solvents, and low-energy milling, which makes the ink formulation promising. Furthermore, we report a simple and easy method for introducing sodium solutions by spin-coating deposition process into the absorber layer in terms of crystallinity, thickness, and grain growth, which is desirable for absorber layers in the photovoltaic application. Considering these improvements of our suggested method for ink fabrication and the encouraging results of deposited thin films, nano-powder ink fabrication has shown to be more efficient for bulk production than the other conventional methods reported in the literature. 

A mechanochemical method was used to synthesize CZTS nanoparticles with kesterite structures. These nanoparticles were used in ink formulations. The deposition of thin films with and without Na solution was carried out using the spin-coating process, followed by annealing at different temperatures in a furnace under an N_2_ environment. The prepared CZTS thin films, with and without the Na solution, were characterized using several techniques. The solar cell application of the CZTS devices was analyzed based on the J–V characteristics.

## 2. Materials and Methods

### 2.1. Mechanochemical Fabrication of CZTS Nanopowder and Ink Preparation

**Figure 1:**
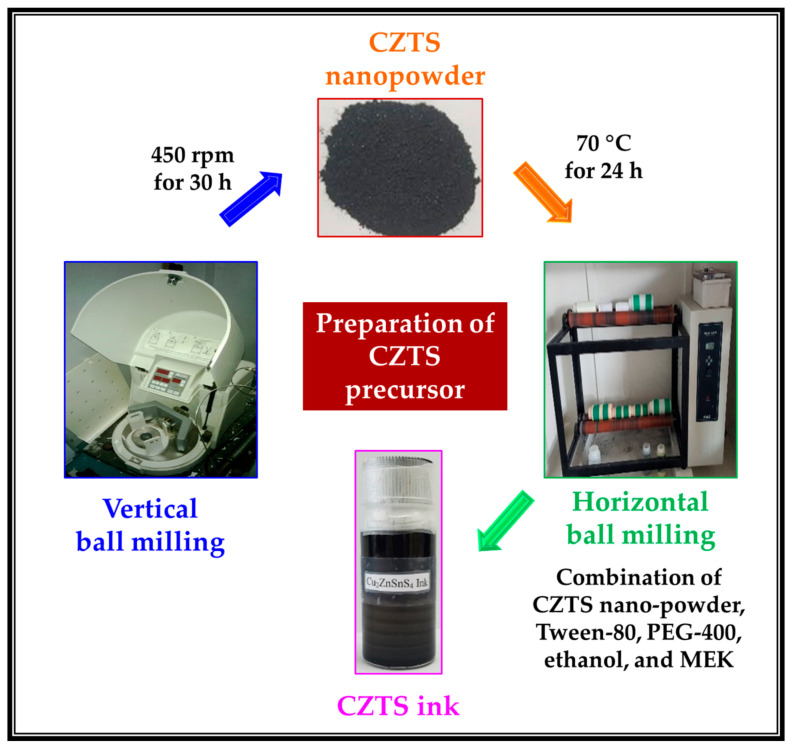
Vertical and horizontal ball milling for CZTS nanopowder and ink synthesis.

CZTS nanopowders and inks were prepared by a mechanochemical method. For the CZTS synthesis, Cu, Zn, Sn, S, and butanol were placed in an 80 mL bowl and ball-milled at 450 rpm for 30 h to obtain a homogenous mixture of single-phase CZTS nanopowder. The as-synthesized homogeneous mixture was dried in an oven at 70 °C for 24 h. The prepared nanopowders were used for ink formulation (Figure 1).

For the synthesis of the CZTS ink, the nanoparticles were ground using an agate motor, the ethanol and MEK solvent mixture was added, and then ultrasonicated for 1 h. After ultrasonication, the surfactant (Tween-80) and binder (PEG-400) were added and milled for >48 h to obtain a stable ink. The prepared ink is denoted CZTS-Ink. To control the film thickness, the prepared ink was diluted (ink:solvent, 1:1, Figure 1). 

### 2.2. Preparation of Na Solution

Sulfur powder (1.0 mmol) was added to a vial containing a NaBH_4_ (1.2 mmol)/C_2_H_5_OH (25 mL) solution and stirred at 75 °C for 15 min, to prepare a Na solution. 

### 2.3. CZTS Thin-Film Coating, Annealing, and Photovoltaic Device Fabrication

As-formulated CZTS inks were used to fabricate thin films via a two-step spin-coating process. The CZTS inks were spun on Mo-coated SLG substrates at 2500 rpm for 30 s and then at 3500 rpm for 30 s, followed by heating on a hotplate in the air for 1 min at 75 °C. The spin-coating step was repeated three times, and each layer of the thin film was heated to obtain the desired thickness. To prepare samples with Na, two layers of Na solution were spin-coated on the CZTS absorber layer (Figure S1).

The deposited CZTS thin films, with and without a Na layer, were annealed at different temperatures (Figure S1c). The thin films were loaded in a graphite box with elemental S powder and annealed at different temperatures (500, 520, and 550 °C) in a tube furnace for 30 min under continuous N_2_ flow. The annealing temperatures are selected in a range in which CZTS does not decompose while it is fully crystallized. The tube was purged with the same gas for 15 min before annealing to remove the air. After heat treatment and before the final collection of the film, the furnace was allowed to cool naturally to room temperature. 

The prepared CZTS thin films were integrated into photovoltaic devices in the traditional substrate configuration with and without Na, i.e., Mo/CZTS:Na orCZTS/CdS/ZnO/ZnO:Al/Ni-Al and. A CdS buffer layer (approximately 50 nm) was fabricated onto the CZTS film by the chemical bath deposition method. Intrinsic ZnO (50 nm) and ZnO:Al (400 nm) window layers were sputtered onto the buffer layer using RF magnetron sputtering. Finally, a Ni/Ag grid (50 and 1600 nm) was fabricated by e-beam evaporation onto the window layer to form a front contact. 

The as-deposited thin films with and without Na solution were denoted S0_Na and S0, respectively; the thin films annealed at 500, 520, and 550 °C with Na solution were denoted S1_Na, S2_Na, and S3_Na, respectively; those annealed at 500, 520, and 550 °C without Na were denoted S1, S2, and S3, respectively. The as-fabricated photovoltaic devices, using the annealed thin films, were denoted SC-S1, SC-S2, SC-S3, SC-S1_Na, SC-S2_Na, and SC-S3_Na.

## 3. Results and Discussion

### 3.1. XRD Analysis 

**Figure 2:**
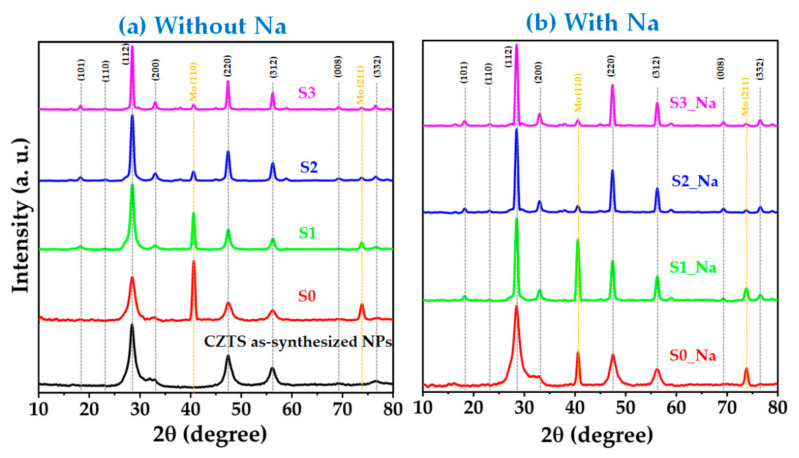
X-ray diffraction patterns of CZTS thin films (**a**) without and (**b**) with Na layer.

The XRD diffraction patterns of the as-synthesized CZTS thin films (S0, S1, S2, S3, S0_Na, S1_Na, S2_Na, and S3_Na) are presented in Figure 2. Sharp XRD diffraction peaks were detected around 2*θ* = 28.54°, 47.57°, and 56.40°, correlated with d-spacings of 3.133, 1.916, and 1.636 Å, which were attributed to the diffraction planes of (112), (220), and (312) of the CZTS kesterite structure (JCPDS card no. 26-0575). In addition, several weak peaks corresponding to the (101), (110), (200), (008), and (332) diffraction planes appeared in the XRD spectra of the annealed CZTS thin films. Along with the peaks of the CZTS absorber, XRD peaks for Mo were also observed around 2*θ* = 40.53° (110) and 73.78° (211). The relative peak intensities of all annealed CZTS thin films increased due to an increase in crystallinity by thermal annealing, while the unannealed samples revealed a low peak intensity with an amorphous nature. No noticeable XRD peaks representing the binary and/or ternary phases were detected in the CZTS thin films with and without Na, which validates the formation of a single kesterite phase in all CZTS thin films. 

The structural parameters of the CZTS samples were calculated using their XRD diffraction patterns. The average crystallite size of the prepared CZTS samples was calculated using the Debye–Scherrer formula, as follows [31]:

(1) $$D=\frac{k\;\lambda }{\beta_{d}\;cos\theta }$$

where *D* is crystallite size in nanometers, $\beta_{d}$ is full width at half maximum, *λ* is wavelength of the incident radiation (Cu-K_α_ = 0.15406 nm), *k* is shape factor (0.94), and *θ* is diffraction angle. The lattice parameters for tetragonal CZTS samples can be estimated from the following equation:

(2) $$\frac{1}{d_{hkl}^{2}}=\frac{h^{2}+k^{2}}{a^{2}}+\frac{l^{2}}{c^{2}}$$

where *d_hkl_* is the interplanar distance; *h*, *k*, and *l* are the Miller indices; and a and c are the lattice parameters. 

Bragg’s diffraction angles (2*θ*) selected for lattice constant estimation were 28.54° and 56.4°, which corresponded to the (112) and (312) diffraction planes, respectively.

The microstrain induced by crystal distortion and imperfections in the thin film prepared by nanoparticle ink can be determined using the following equation:

(3) $$\varepsilon =\frac{\beta_{d}}{4tan\theta }$$

From Equation (1), it can be seen that the Debye–Scherrer method shows a dependence on 1/*cosθ*, whereas the Williamson–Hall analysis varies with *tanθ*. This elemental difference allows for the separation of broad peaks, for two reasons relating to the microstructure, i.e., the microstrain and small crystallite size, which simultaneously occur in the sample. We assume that both the components, i.e., crystallite size and microstrain, contribute to the total integral broadening of the peak. Thus, the sum of Equations (1) and (3) yields the following relationship:

(4) $$\beta_{d}=\frac{k\lambda }{D\;cos\theta }+4\varepsilon \;tan\theta$$

In Equation (4), multiply the *cosθ* of both the sides, and one derives:

(5) $$\beta_{d}cos\theta =\frac{k\lambda }{D}+4\varepsilon \;sin\theta$$

Equation (5) describes the Williamson–Hall method. The number of defects in the crystal structure is determined by calculating the dislocation density (δ) [31], which is estimated using the following formula:

(6) $$\delta =\frac{1}{D^{2}}$$

The calculated values of the structural parameters are summarized in Table 1. These estimated values are in good concordance with the lattice parameters of standard JCPDS card no, 26-0575, *a* = *b* = 5.43 Å and *c* = 10.86 Å. A tetragonal distortion value, that is, the *c*/2*a* ratio of 1, was observed in all the prepared CZTS samples. These results confirm that the kesterite structure is consistent with those from previously published reports [22,32]. The average crystallite size was calculated to be 6–109 nm using the Debye–Scherrer equation, whereas the average crystallite size obtained from Williamson–Hall measurements was between 5 and 61 nm. An increase in the average crystallite size with annealing temperature indicates an enhanced crystal quality of the absorber material. An increase in the microstrain is caused by the stretching or compression of the lattice towards the c-axis direction [33,34].

The dislocation density refers to the number of imperfections, that is, defects and vacancies, in the crystal structure [35]. As shown in Table 1, a decrease in the dislocation density with increasing temperature is observed. These results further suggest an improvement in the crystalline quality of the CZTS samples with increasing temperature. Notably, the XRD diffraction peaks for CZTS overlap with those of Cu_2_S, ZnS, and Cu_2_SnS_3_; therefore, XRD results are not sufficient to attest the crystallization of CZTS; thus, Raman analysis was additionally employed to investigate the phases of the samples.

### 3.2. Raman Analysis

**Figure 3:**
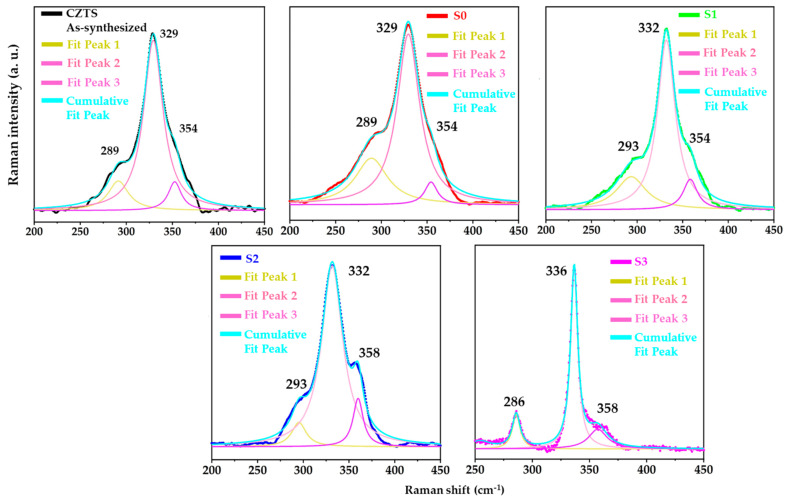
Raman spectra of CZTS thin films.

The Raman spectra were recorded to validate the phase purity of all prepared CZTS thin films (Figure 3). The characteristic Raman peaks of all the prepared CZTS thin films were observed at approximately 289, 329, and 354 cm^−1^. The broadening and shift of the Raman peak positions in the thin films were attributed to the crystallinity and internal lattice stress [11,36].

The most intense vibrational band at 336 cm^−1^ with weak shoulder peaks at 286 and 358 cm^−1^ was ascribed to the CZTS kesterite phase. The strongest band at 336 cm^−1^ was attributed to the A1 symmetry mode, which was ascribed to the vibration of sulfur atoms in the lattice [37]. These observed vibration peaks are consistent with the previously reported Raman peaks of CZTS material [38,39,40]. The Raman peaks for other secondary phases, such as binary and/or ternary phases, have been described in various studies; their vibrational bands are listed in Table S1. The characteristic peaks of the binary and/or ternary phases were not reflected in the Raman analysis of all CZTS thin films, which validated the formation of a single-phase in each synthesized CZTS thin film. The Raman analysis demonstrated a good agreement with the XRD results.

### 3.3. FT-IR Analysis

**Figure 4:**
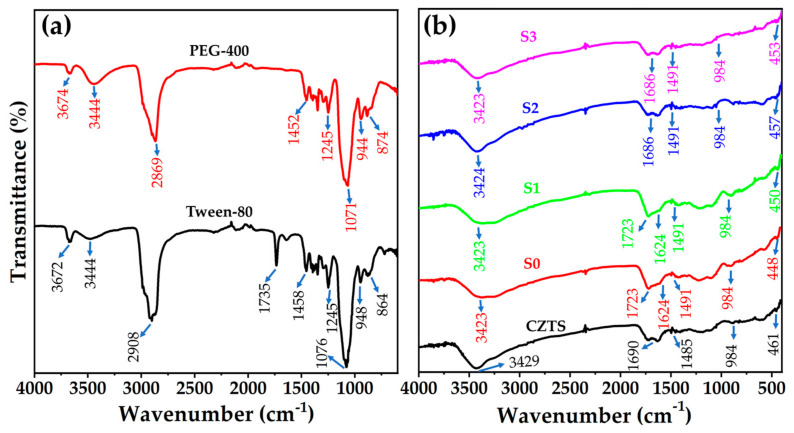
FT-IR spectra of (**a**) Tween-80 and polyethylene glycol (PEG-400) and (**b**) CZTS thin films.

FT-IR spectra of the CZTS samples, PEG-400, and Tween-80 are displayed in the 400–4000 cm^−1^ range (Figure 4). Major FT-IR peaks were observed at approximately 3300, 1723, 1600, 1491, and 984 cm^–1^ for S0 and S1. In addition, a weak absorption band representing metal sulfide can be observed in the 500–400 cm^–1^ range for samples S0 and S1 [41,42]. The FT-IR spectra of pure PEG-400 [43,44] and Tween-80 [45,46] revealed peaks at approximately 3600, 2900, 1070, 940, and 800 cm^–1^ (Figure 4a) The peaks at ~3300 cm^−1^ are ascribed to O–H stretching. The band observed at approximately 2900 cm^–1^ was attributed to the C–H stretching vibrations of the –CH_2_ group. The band at 1245 cm^–1^ was attributed to the C–O stretching vibration. The absorption at 1452 cm^–1^ was designated to the C–H bending vibrations of the –CH_2_ group; however, asymmetrical bending vibrations of –CH_3_ also exist. The peak in the region of approximately 900 cm^–1^ was assigned to C–O–C symmetrical stretching. A characteristic peak at 1735 cm^–1^ related to the C=O group was observed for Tween-80. The band at approximately 600 cm^–1^ was assigned to the ZnS phase and was absent in the S0 and S1 samples. The spectrum for the S0 sample reveals absorption bands in regions similar to those of Tween-80 and PEG-400. This may be because the solvents, binder, and surfactant used for the ink have very similar types of functional bonds, and as a result, the absorption bands are indexed to similar regions.

Major peaks were observed at 3429, 1686, 1485, 984, and 461 cm^–1^ for the as-synthesized samples. The FT-IR peaks in a similar region for the S2 and S3 samples were observed at approximately 3400, 1600, 1491, 984, and 400 cm^–1^. These peaks match those of the synthesized peaks. A weak metal sulfide band can also be observed in the 550–400 cm^–1^ region for as-synthesized, S2, and S3 samples [41,42]. However, the characteristic peaks around 600 cm^–1^ for the ZnS band [37] were not identified in the prepared CZTS samples, confirming the absence of secondary phases. The peaks at approximately 3400 cm^–1^ were ascribed to the S-rich composition. The band at approximately 1600–900 cm^–1^ was attributed to the bending and stretching vibrational frequencies of O=O. The peak at 1452 cm^–1^ was ascribed to the C–H bending vibrations of the –CH_2_ group; however, asymmetrical bending vibrations of –CH_3_ also exist. These bands arise because CZTS is a hygroscopic material, and surface-adsorbed moisture, that is, H_2_O and CO_2_ molecules, contribute to O–H- and C–O-related peaks in the FT-IR spectrum [37]. The FT-IR analysis confirmed the presence of a pure phase in all prepared CZTS samples.

### 3.4. FE-SEM and EDS Analyses

**Figure 5:**
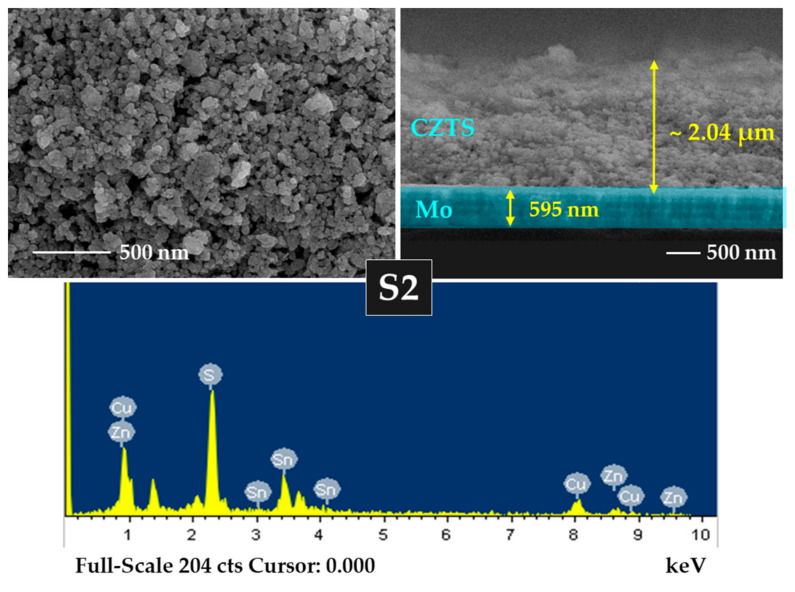
Surface and cross-section images and energy-dispersive X-ray spectroscopy (EDS) spectrum of S2 thin film.

An FE-SEM analysis was performed to analyze the impacts of annealing and Na incorporation on CZTS morphology. Figures S2 and S3 show the FE-SEM images of the S0 and S0-Na thin films, respectively. The surface images show that the S0 and S0-Na thin films contained a mixture of nanoparticles and organic (PEG-400 and Tween-80) molecules. This may be because the earlier heating process was not sufficient to evaporate the organic content used for the ink formulation for an effective spin coating. The optimum temperature for the evaporation of these organic molecules may be greater than 250 °C [47,48]. FE-SEM images of the S1, S2, and S3 thin films are presented in Figure S4 and Figure 5 and Figure S5, respectively. During the annealing process, the organics could be thermally decomposed and slowly evaporated by increasing the temperature from 500 to 550 °C. However, some voids with smaller grain sizes can be seen on the surface of the thin films prepared using the above annealing process. The porous nature of the CZTS thin film prepared using nanoparticle inks has also been reported by other researchers [49,50]. Smaller grains are responsible for the low carrier lifetime and, hence, affect the efficiency of the solar cell [51]. The thicknesses of S0, S1, S2, and S3 thin films were measured to be 2.36, 2.30, 2.04, and 1.85 µm, respectively.

**Figure 6:**
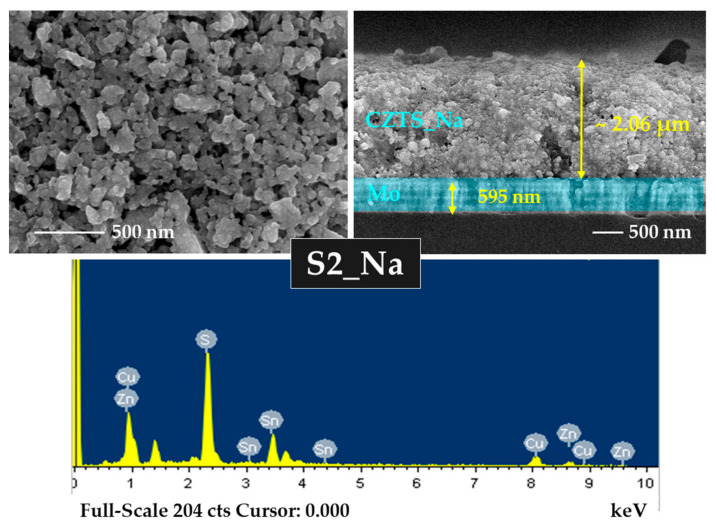
Surface and cross-section images and EDS spectrum of S2_Na thin film with Na layer.

The effect of Na on the morphology of S1-Na, S2-Na, and S3-Na thin films is studied using FE-SEM, as shown in Figure S6, Figure 6 and Figure S7, respectively. The results revealed that the S1-Na, S2-Na, and S3-Na thin films are non-uniform, porous, and vary in grain size. Although the surface morphology of the CZTS:Na thin films is uneven and non-compact, it is conducive to the evaporation of organic materials and the reduction in carbon residues at the bottom of thin films [52]. The thicknesses of S0-Na, S1-Na, S2-Na, and S3-Na thin films were found to be 2.36, 2.14, 2.06, and 1.96 µm, respectively. Notably, relatively large grains are beneficial for enhancing the photovoltaic performance, as such large grains increase the diffusion length of minority carriers and reduce the recombination rate of the light-induced carriers [52,53].

A chemical composition analysis was performed using EDS for all CZTS samples, and the findings are presented in Table S2 and Table 2. The Zn and Sn content in the CZTS samples declined with increasing temperature owing to the thermal evaporation of the elements. The S/metal ratio increased as the annealing temperature increased, indicating substantial incorporation of S into the thin films. However, the ratios of Cu/(Zn + Sn) and Zn/Sn in all CZTS samples reveal a relatively small deviation from their stoichiometric values.

### 3.5. Optical Properties

A measurement of the optical characteristics of semiconductor compounds is important for determining their potential use in photovoltaic applications. The bandgap of the thin films was estimated by plotting (*αhv*)*^x^* vs. *hv*(eV), as in Equation (7), and extrapolating the linear region of the spectrum in the high-absorption regime, where the intercept with the photon energy axis yields the bandgap as follows [54]:

(7) $$\alpha hv=A{\left(hv-E_{g}\right)}^{x}$$

where *α* is an absorption coefficient, *hv* is an incident photon energy (eV), *A* is a constant, *E_g_* is an optical bandgap energy (eV), and *x* is a constant depending on the optical transition, that is, the direct or indirect transition of the semiconductor materials. The transmission spectra of the semiconductor thin films were recorded using a UV-Vis-NIR spectrophotometer. Using transmission data, the absorption coefficient (*α*) was estimated by employing the following relationship [49]:

(8) $$\alpha =\frac{1}{d}\ln\left(\frac{1}{T}\right)$$

where *d* is the thin-film thickness and *T* is the transmission spectrum of the semiconductor thin films.

**Figure 7:**
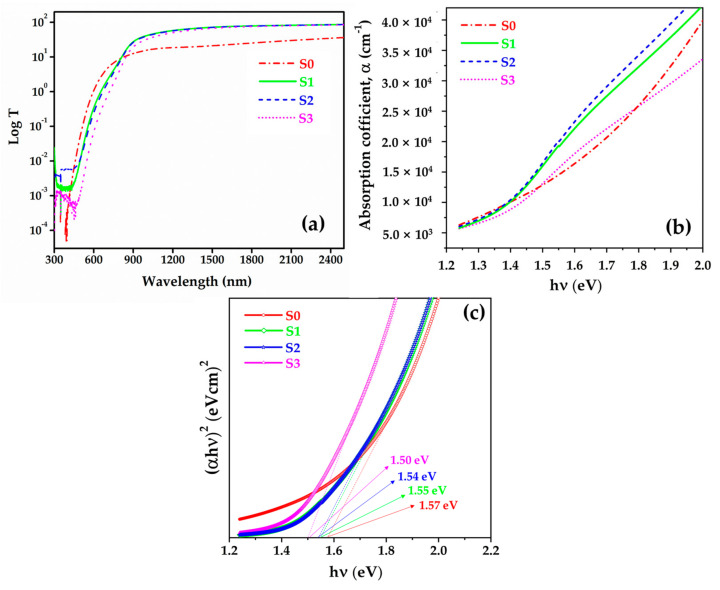
(**a**) Log transmission spectra, (**b**) absorption coefficient spectra, and (**c**) bandgap of CZTS thin films.

The optical properties of the prepared CZTS thin films were measured using optical transmission measurements in the range of 300–2500 nm (Figure 7a). Surface morphology is important for influencing the transmittance of CZTS thin films. Information about the surface morphology can explain the variations in the transmission spectra of CZTS thin films annealed at numerous temperatures [54]. Generally, CZTS absorption coefficient in the range of the visible region is higher than 10^4^ cm^−1^ (Figure 7b) [55]. As shown in Figure 7c, the approximate values of the optical bandgap are obtained in the range of 1.50–1.57 eV [56,57]. The bandgap values obtained for the prepared CZTS samples are consistent with those of previous reports [54,56,57].

### 3.6. Electrical Properties

The electrical properties of CZTS samples were measured using Hall measurements, specifically, the Van der Pauw approach, with a magnetic field of 0.55 T at room temperature. For the Hall measurement, CZTS samples were prepared by spin coating on a glass substrate with dimensions of 1 cm × 1 cm. To study the effects of Na on the electrical properties, the prepared Na solution was spin-coated onto CZTS thin films. All prepared CZTS thin films were annealed at numerous temperatures. For the metal contact, the Ag paste was applied to the corners of the thin films. All CZTS thin films exhibited p-type conductivity. The resistivity, mobility, and carrier density or concentration of the CZTS samples are summarized in Table 3.

The carrier density of the CZTS samples was between 4.17 × 10^17^ and 1.53 × 10^18^ cm^−3^, which is consistent with reported values (10^16^ cm^−3^ to 10^19^ cm^−3^) [58,59,60]. The mobility of the CZTS samples was between 2.201 and 3.559 cm^2^ V^–1^ s^–1^, which is consistent with that of other reported thin films (1–10 cm^2^ V^–1^ s^–1^) [3,59]. As shown in Table 3, on increasing the annealing temperature, the resistivity increases, and the carrier density decreases. Samples S1 and S1-Na revealed relatively high carrier densities and mobilities with low resistivities. The presence of carbon content in samples S1 and S1-Na may explain the high mobility and carrier density with a low resistivity [61]. However, low mobility and carrier density with high resistivity were observed in the annealed samples. This may be due to the presence of voids, small grain sizes, and the porous nature of the thin films [62]. Although the present values are less consistent with reported values for mobility (0.1–10 cm^2^ V^–1^ s^–1^), carrier density (10^16^–10^20^ cm^−3^), and resistivity (10^−3^–9.7 Ω cm) for CZTS samples [36,63,64], there is room for optimization.

The structural, phase purity, surface morphology, compositional, optical, and electrical properties indicate that CZTS thin films fabricated by the ink method can be used as an absorber layer for thin-film solar cells.

### 3.7. Photovoltaic Performance

**Figure 8:**
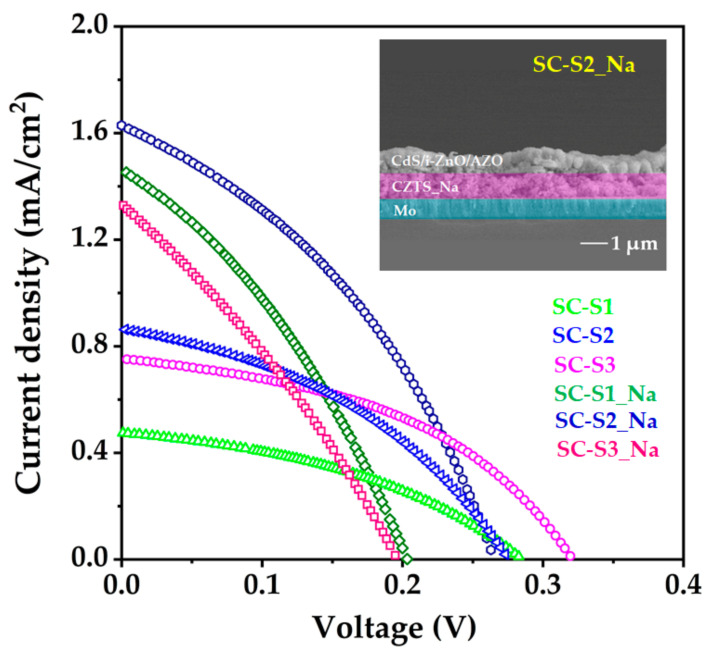
J–V curve of CZTS photovoltaic device (S2_Na) with an inset of the cross-sectional image.

CZTS absorber layers with and without Na were integrated into the photovoltaic device in the substrate configurations of SLG/Mo/ CZTS:Na or CZTS/CdS/i-ZnO/ZnO:Al/Ni-Ag. J–V curves of the photovoltaic devices are shown in Figure 8. The total active area of each photovoltaic device is 0.40 cm^2^. The performance parameters of the fabricated photovoltaic devices are tabulated in Table 4. The outcomes in Figure 8 and Table 4 reveal that photovoltaic devices based on CZTS thin films with a Na layer, which were annealed at low temperatures (S1_Na and S2_Na), demonstrated improvements in power conversion efficiency. This may be due to the improved crystallinity of CZTS, which reduces the defects at the grain boundaries. In the photovoltaic devices based on CZTS thin films with and without Na at different temperatures, samples S1 and S3_Na demonstrated poor performance, possibly due to pinholes, roughness, and many voids present on CZTS thin-films surface, as mentioned above. The presence of pinholes and voids typically leads to shunting problems in the device. Furthermore, voids present on the CZTS thin-film surface may result in further defects and increased internal resistance, which can decrease the performance of the PV device. Therefore, there is a slight difference in performance between samples S2 and S3, possibly due to the voids formed by the uneven grain growth. Finally, sample S2_Na exhibited the best result among all the devices, with an efficiency of 0.16%, a J_sc_ of 1.61 mA cm^–2^, an FF of 37.48%, and a V_oc_ of 274 mV. However, the power conversion efficiency is far lower than the reported conversion efficiency of a CZTS-based photovoltaic device (9.2% to 11.0%) using an absorber fabricated by a vacuum-based technique (evaporation, co-sputtering, and RF-magnetron sputtering) [65,66]. This could be because all the CZTS samples contain small-sized grains [49,67,68], possibly because of the non-optimized process of sulfurization [49,67,68] and Na incorporation [22,23]. Zn-rich and Cu-poor compositions (Zn/Sn = 1.2 and Cu/(Zn + Sn) = 0.8) are ideal conditions for high conversion efficiency; thus, the elemental composition of CZTS samples requires optimization [49,69,70]. Further study is being carried out in our laboratory to enhance the performance of photovoltaic devices. The inset in Figure 8 shows a cross-sectional image of the photovoltaic device that incorporates sample S2_Na.

## 4. Conclusions

In summary, a simple, non-toxic, and cost-effective mechanochemical process was adopted to fabricate CZTS absorber materials and photovoltaic devices. The effects of various annealing temperatures (500–550 °C) on the phase purity, morphology, elemental composition, and optoelectronic properties of the fabricated CZTS samples were examined. XRD, Raman, and FT-IR analyses validated the formation of the kesterite CZTS phase. The EDS analysis demonstrated a substantial incorporation of S into the absorber films during the annealing at various temperatures, and optical bandgaps were obtained in the range of 1.50–1.57 eV. The carrier concentration, resistivity, and mobility of sample S2_Na were measured to be 9.15 × 10^17^ cm^−3^, 2.113 Ω cm, and 3.229 cm^2^ V^–1^ s^–1^, respectively. The photovoltaic efficiency of the device fabricated from S2_Na was 0.16%. Future research will focus on optimizing the temperature profile of the sulfurization process, to obtain a larger grain size without pores, and forming homogeneous CZTS thin films to fabricate high-efficiency CZTS photovoltaic devices.

## Figures and Tables

**Table 1:** Structural parameters: crystallinity (*D*), strain (*ε*), dislocation density (δ), and lattice constant (*a* = *b* and *c*) of CZTS thin film annealed at different temperatures.

Parameter		Sample Name				
		CZTS As-Synthesized	CZTS As-Syn. Ink (S0)	CZTS-500 (S1)	CZTS-520 (S2)	CZTS-550 (S3)
Phase		Kesterite				
Scherrer	*D* (nm)	6.34	7.47	16.21	24.81	108.54
	*δ* (nm^−2^)	0.02480	0.01787	0.00380	0.00162	0.00008
Williamson–Hall	*D* (nm)	5.39	5.30	8.68	19.75	60.28
	*ε* (× 10^−3^)	−4.7	−6.7	−5.5	−1.5	−0.7
	*δ* (nm^−2^)	0.03436	0.03552	0.01327	0.00256	0.00028
Lattice Constant	*a* (Å)	5.42	5.42	5.42	5.42	5.42
	*c* (Å)	10.86	10.86	10.87	10.87	10.87
	*c*/2*a*	1.0007	1.0003	1.0033	1.0031	1.0021
Lattice structure		tetragonal				

**Table 2:** Elemental composition of CZTS with and without Na layer.

Sl. No	Sample Name	Cu/(Zn + Sn)	Zn/Sn	S/Metal
1	S0	1.06	1.15	1.28
2	S1	0.97	1.41	0.82
3	S2	0.95	1.20	0.81
4	S3	1.03	1.01	0.92
5	S0_Na	1.20	1.03	1.52
6	S1_Na	1.06	1.04	0.84
7	S2_Na	1.02	0.95	0.95
8	S3_Na	1.13	0.98	1.04

**Table 3:** Electrical properties of CZTS thin films with and without Na layer.

Sl. No.	Sample Name	Carrier Conc. (cm^−3^)	Mobility (cm^2^/V.s)	Resistivity (Ω cm)
1	S1	1.53 × 10^18^	3.55	1.145
2	S2	1.14 × 10^18^	2.39	2.294
3	S3	6.83 × 10^17^	2.40	3.798
4	S1_Na	1.51 × 10^18^	3.28	1.257
5	S2_Na	9.15 × 10^17^	3.22	2.113
6	S3_Na	4.17 × 10^17^	2.20	6.804

**Table 4:** Photovoltaic properties of CZTS thin films with and without Na layer.

Sl. No.	Sample Name	V_oc_ (mV)	J_sc_ (mA/cm^2^)	FF (%)	η (%)
1	SC-S1	280	0.48	38.64	0.05
2	SC-S2	275	0.85	41.42	0.09
3	SC-S3	320	0.74	44.25	0.10
4	SC-S1_Na	201	1.47	35.16	0.10
5	SC-S2_Na	274	1.61	37.48	0.16
6	SC-S3_Na	198	1.33	34.13	0.09

## Data Availability

Not applicable.

## Supplementary Materials

The following supporting information can be downloaded at: https://www.mdpi.com/article/10.3390/ma15051708/s1. Figure S1: Spin-coating process for deposition of Cu_2_ZnSnS_4_ thin films (a) without and (b) with sodium solution, (c) Annealing profile of Cu_2_ZnSnS_4_ thin films; Figure S2: Surface and cross-section images and EDS spectra of S0 thin film; Figure S3: Surface and cross-section images and EDS of S0_Na thin film with sodium layer; Figure S4: Surface and cross-section images and EDS spectra of S1 thin film; Figure S5: Surface and cross-section images and EDS spectra of S3 thin film with sodium layer; Figure S6: Surface and cross-section images and EDS spectra of S1_Na thin film; Figure S7: Surface and cross-section images and EDS spectra of S3_Na thin film with sodium layer; Table S1: Raman scattering band position of Cu_2_ZnSnS_4_ and other secondary phases; Table S2: Elemental composition of Cu_2_ZnSnS_4_ with and without sodium layer.
