# Supplementary material for: Fabrication of Cu2ZnSnS4 Light Absorber Using a Cost-Effective Mechanochemical Method for Photovoltaic Applications
Source: Materials (Basel). 2022 Feb 24;15(5):1708. doi: 10.3390/ma15051708 (PMC8911092; doi:10.3390/ma15051708)
Supplement: Supplementary file 1 [file materials-15-01708-s001.zip › materials-1591455-supplementary.pdf]

materials

MDPI

Supplementary Material

Fabrication of Cu_2ZnSnS_4 Light Absorber Using a Cost-Effective Mechanochemical Method for Photovoltaic Applications

Meenakshi Sahu [1,2], Vasudeva Reddy Minnam Reddy [3], Bomyung Kim [3], Bharati Patro [4], Chinho Park [2,*], Woo Kyoung Kim [3,*] and Pratibha Sharma [1,*]

1 Department of Energy Science and Engineering, Indian Institute of Technology Bombay Powai, Mumbai 400076, India; meenakshisahu.chem@gmail.com
2 Korea Institute of Energy Technology (KENTECH), 200 Hyukshin-ro, Naju, Jeollanam-do 58330, Korea
3 School of Chemical Engineering, Yeungnam University, Gyeongsan 38541, Korea; drmvasudr9@gmail.com (V.R.M.R.); billionp10@ynu.ac.kr (B.K.)
4 Centre for Research in Nanotechnology and Sciences Indian Institute of Technology Bombay Powai, Mumbai 400076, India; bharati@iitb.ac.in
* Correspondence: chpark@kentech.ac.kr (C.P.); wkim@ynu.ac.kr (W.K.K.); pratibha_sharma@iitb.ac.in (P.S.)

Contents:

Characterization
Figure S1: Spin-coating process for deposition of Cu_2ZnSnS_4 thin films.
Figure S2: Surface and cross-section images and EDX spectra of S0 thin film.
Figure S3: Surface and cross-section images and EDX of S0_Na thin film with sodium layer.
Figure S4: Surface and cross-section images and EDX spectra of S1 thin film.
Figure S5: Surface and cross-section images and EDS spectra of S3 thin film with sodium layer .
Figure S6: Surface and cross-section images and EDS spectra of S1_Na thin film.
Figure S7: Surface and cross-section images and EDS spectra of S3_Na thin film with sodium layer;
Table S1: Raman scattering band position of Cu_2ZnSnS_4 and other secondary phases
Table S2: Elemental composition of Cu_2ZnSnS_4 without and with sodium layer

Citation: Sahu, M.; Minnam Reddy, V.R.; Kim, B.; Patro, B.; Park, C.; Kim, W.K.; Sharma, P. Fabrication of Cu_2ZnSnS_4 Light Absorber Using a Cost-Effective Mechanochemical Method for Photovoltaic Applications. *Materials* **2022**, *15*, 1708. https://doi.org/10.3390/ma15051708

Academic Editor: Nikolas J. Podraza

Received: 25 January 2022
Accepted: 22 February 2022
Published: 24 February 2022

Publisher's Note: MDPI stays neutral with regard to jurisdictional claims in published maps and institutional affiliations.

Copyright: © 2022 by the authors. Licensee MDPI, Basel, Switzerland. This article is an open access article distributed under the terms and conditions of the Creative Commons Attribution (CC BY) license (https://creativecommons.org/licenses/by/4.0/).

Characterization

The prepared CZTS thin films were analyzed using various characterization techniques, such as X-ray diffraction (XRD), Raman spectroscopy, field-emission scanning electron microscopy (FE-SEM), energy-dispersive X-ray spectroscopy (EDS), Fourier-transform infrared (FT-IR) spectroscopy, ultraviolet-visible near-infrared (UV-Vis-NIR) spectroscopy, and Hall measurements. The crystallographic information of the as-fabricated and annealed thin films was evaluated using an X-ray diffractometer (PANalytical X′ Pert-PRO MPD) with monochromatized Cu Kα radiation (λ = 1.5406 Å). The XRD patterns were recorded in the 2θ range of 10°–80° at a 4°/min scanning rate and a step size of 0.02°. Phase analysis of the CZTS samples was carried out using a Jobin-Yvon-Horiba (model no-HR800UV) Raman spectrometer at room temperature with a 514.5 nm laser and a working power of 10 mW. The surface morphology and cross-sectional structure of the samples were observed using FE-SEM (Hitachi, model no-S-4800). EDS was used to study the chemical composition of the samples. The optical properties of all CZTS thin films were measured in the wavelength range of 300–2500 nm using a UV-Vis-NIR spectrometer (Varian, model no-UV-5000). The electrical properties were investigated using the Hall effect measurement (model no-HMS5000) with the Van der Pauw approach with the current in the range of 1.0–0.1 nA and magnetic field intensity of 0.55 T at room temperature. Ag paste was used to make a metal contact at the corner of the thin film. FT-IR spectra were collected using a PerkinElmer FT-IR spectrometer (model no., Spectrum-100) in the KBr mode. The current density-voltage (J–V) curve of the 0.4 x 0.4 cm^2 fabricated solar cells was measured with a Keithley (model no., 2400). A Xe flash lamp (USHIO, flash type, UA-DF1, 1,000 W, 400 V) with a standard AM1.5 (100 mW cm^{-2}) filter was used as the light source.

Figure S1:

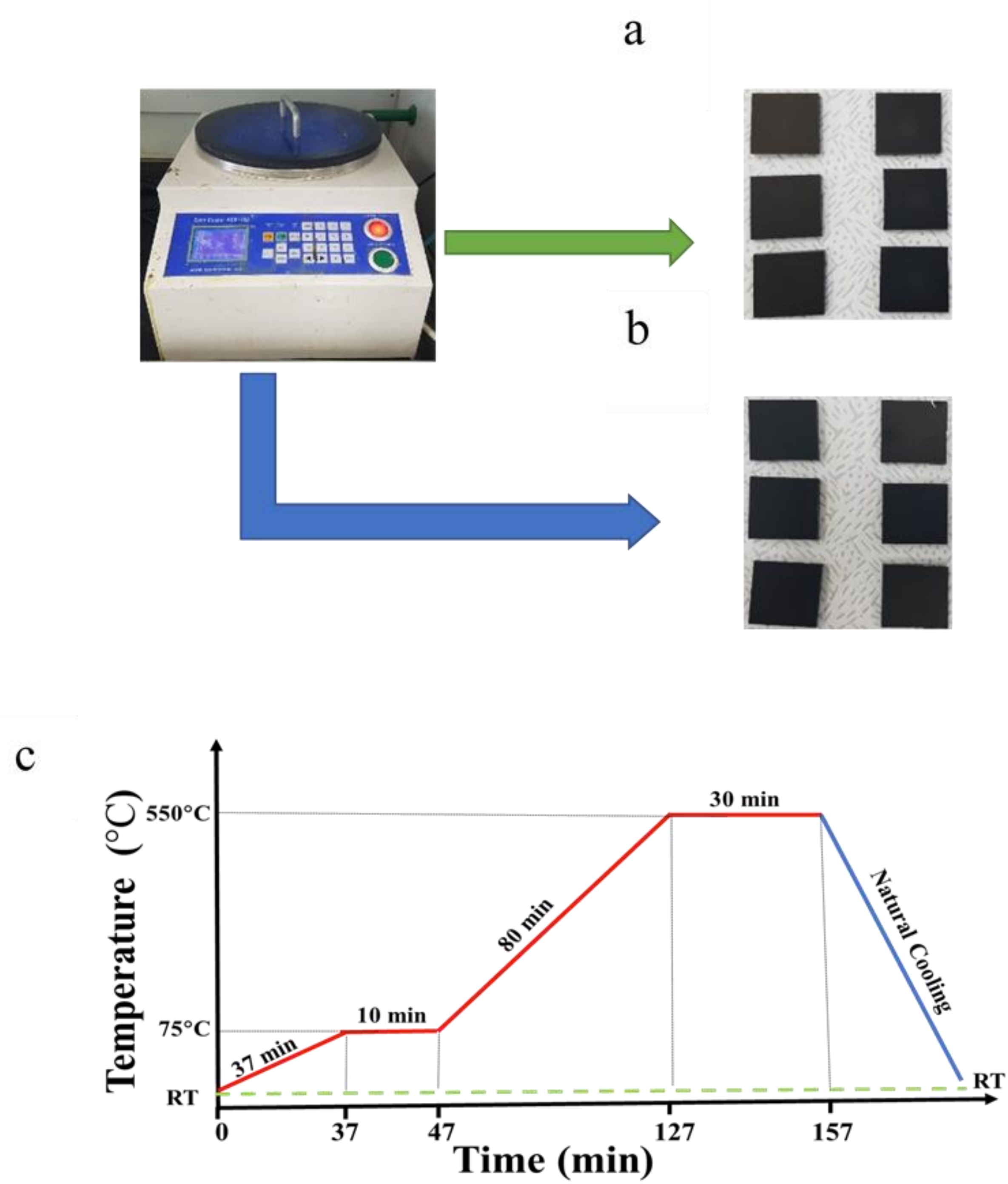

Figure S1. Spin Coating process for deposition of Cu_2ZnSnS_4 thin films (a) without and (b) with a sodium solution, (c) Annealing profile of Cu_2ZnSnS_4 thin films.

Figure S2:

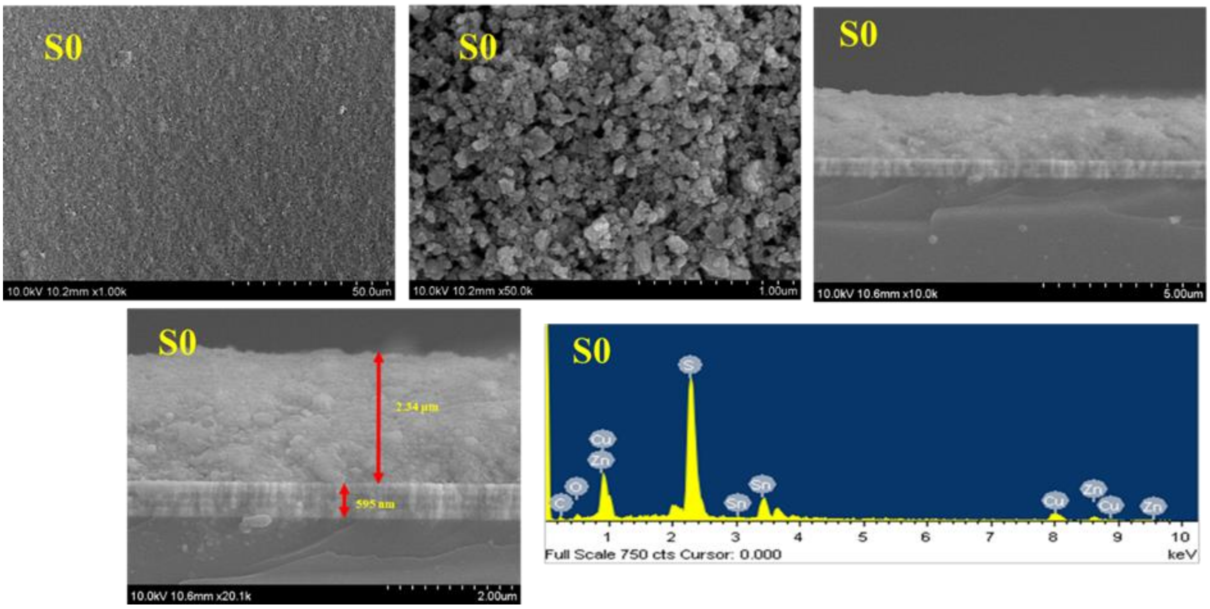

Figure S2. Surface and cross-section images and EDX spectrum of S0 thin film.

Figure S3:

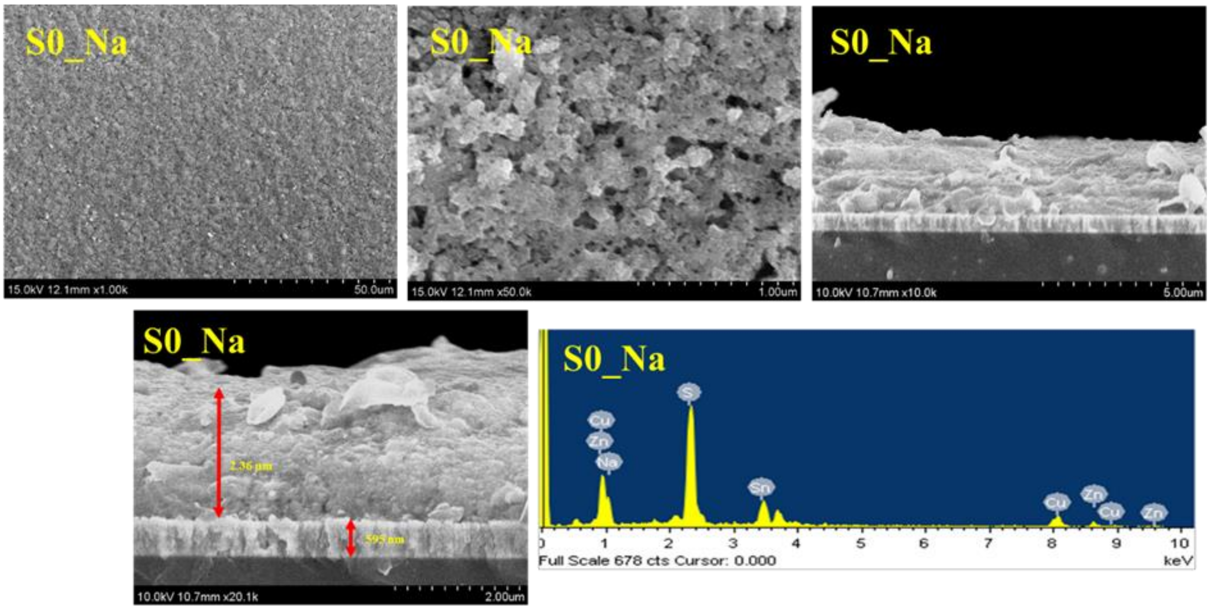

Figure S3. Surface and cross-section images and EDX spectrum of S0_Na thin film with sodium layer.

Figure S4:

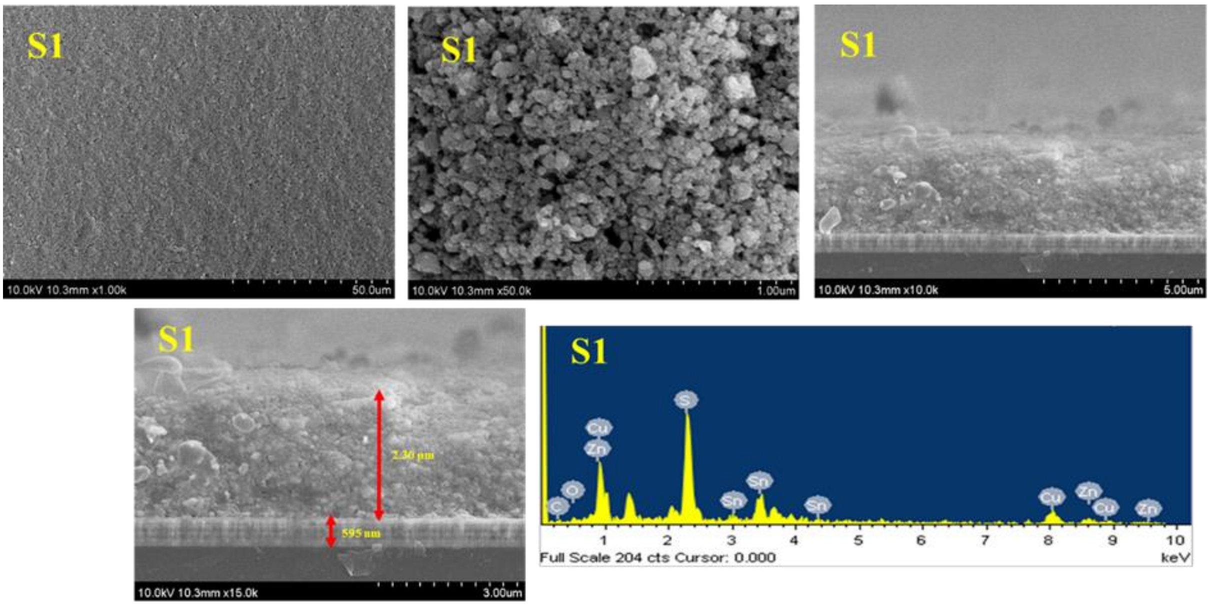

Figure S4. Surface and cross-section images and EDX spectrum of S1 thin film.

Figure S5:

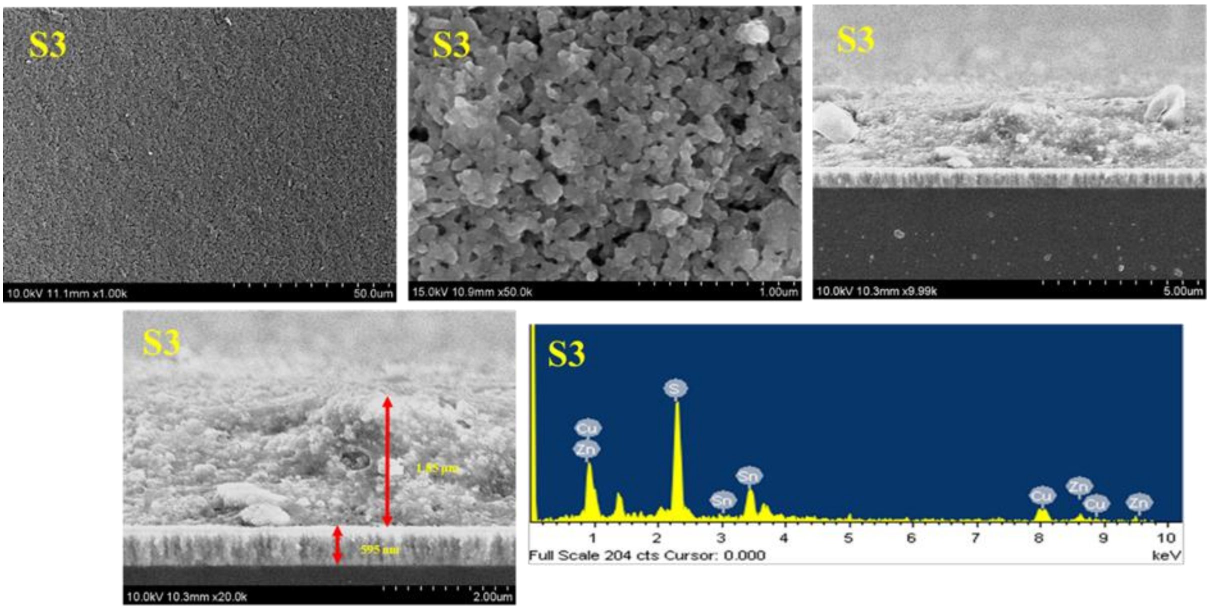

Figure S5. Surface and cross-section images and EDS spectra of S3 thin film with sodium layer

Figure S6:

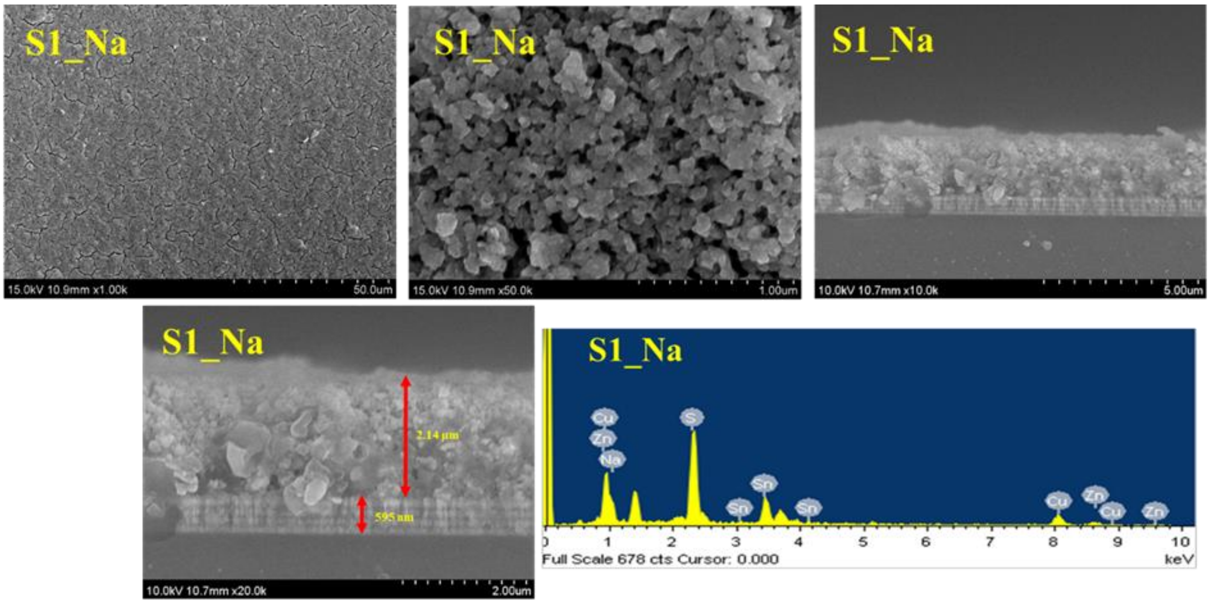

Figure S6. Surface and cross-section images and EDS spectra of S1_Na thin film.

Figure S7:

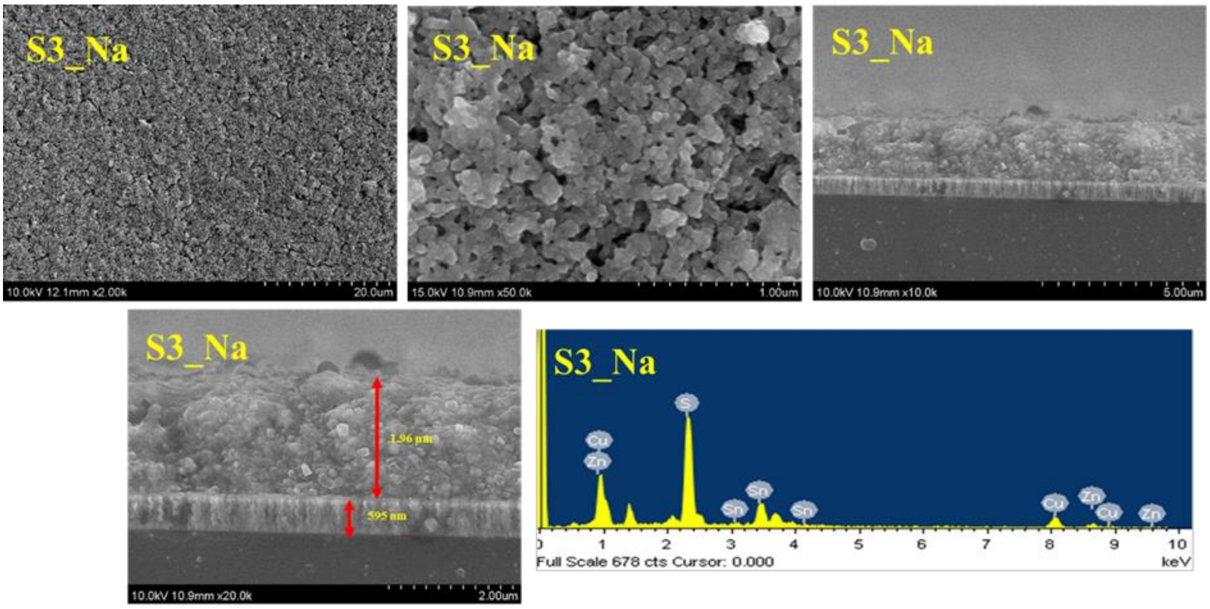

Figure S7. Surface and cross-section images and EDS spectra of S3_Na thin film with sodium layer.

Table S1. Raman scattering band position of Cu_2ZnSnS_4 and other secondary phases.

Sl. No.	Phase	Raman peak position	Ref.
1	Cu_2ZnSnS_4	289, 338, 358, 370	[S1] [S2] [S3] [S4]
2	Hexa-ZnS	355	[S5]
3	Cubic ZnS	348	[S2]
4	$Cu_2Sn_3S_7$	268, 318, 375	[S6]
5	Ort.Cu_3SnS_4	318	[S2]
6	Monoclinic Cu_2SnS_3	290, 352	[S7]
7	Tetrg.Cu_2SnS_3	297, 337, 352	[S2],[S7]
8	Cubic Cu_2SnS_3	267, 303, 356	[S2],[S7]
9	$Cu_{2-x}S$	475	[S8]
10	Sn_2S_3	32,60,307	[S9]
11	SnS_2	315	[S10]
12	SnS	96, 163, 189, 220, 288	[S10]

Table S2. Elemental composition of Cu_2ZnSnS_4 without and with sodium layer.

Sl No	Sample Name	Cu%	Zn%	Sn%	S%	Na%	C%	O%
1	S0	15.70	7.90	6.83	39.11	-	19.37	11.37
2	S1	26.97	16.28	11.52	45.23	-	-	-
3	S2	26.99	15.34	12.78	44.89	-	-	-
4	S3	26.38	12.87	12.73	48.02	-	-	-
5	S0_Na	13.83	5.83	5.61	38.62	3.70	17.45	14.96
6	S1_Na	26.35	12.68	12.09	43.42	5.45	-	-
7	S2_Na	24.26	11.52	12.04	45.51	6.68	-	-
8	S3_Na	24.60	10.78	10.93	48.52	5.16	-	-

Reference:

S1. Indubala, E., et al., *Secondary phases and temperature effect on the synthesis and sulfurization of CZTS.* Solar Energy, 2018. **173**: p. 215-224.

S2. Fernandes, P., P. Salomé, and A. Da Cunha, *Study of polycrystalline Cu_2ZnSnS_4 films by Raman scattering.* Journal of alloys and compounds, 2011. **509**(28): p. 7600-7606.

S3. Dimitrievska, M., et al., *Multiwavelength excitation Raman scattering study of polycrystalline kesterite Cu_2ZnSnS_4 thin films.* Applied Physics Letters, 2014. **104**(2): p. 021901.

S4. Lin, X., et al., *Structural and optical properties of Cu_2ZnSnS_4 thin film absorbers from ZnS and Cu_3SnS_4 nanoparticle precursors.* Thin Solid Films, 2013. **535**: p. 10-13.

S5. Shin, S.W., et al., *Studies on Cu_2ZnSnS_4 (CZTS) absorber layer using different stacking orders in precursor thin films.* Solar energy materials and solar cells, 2011. **95**(12): p. 3202-3206.

S6. Cheng, A.-J., et al., *Imaging and phase identification of Cu_2ZnSnS_4 thin films using confocal Raman spectroscopy.* Journal of Vacuum Science & Technology A: Vacuum, Surfaces, and Films, 2011. **29**(5): p. 051203.

S7. Berg, D.M., et al., *Raman analysis of monoclinic Cu_2SnS_3 thin films.* Applied Physics Letters, 2012. **100**(19): p. 192103.

S8. Emrani, A., P. Vasekar, and C.R. Westgate, *Effects of sulfurization temperature on CZTS thin film solar cell performances.* Solar Energy, 2013. **98**: p. 335-340.

S9. Fernandes, P., P. Salomé, and A. Da Cunha, *Growth and Raman scattering characterization of Cu_2ZnSnS_4 thin films.* Thin solid films, 2009. **517**(7): p. 2519-2523.

S10. Parkin, I., et al., *The first single source deposition of tin sulfide coatings on glass: aerosol-assisted chemical vapour deposition using [Sn (SCH_2CH_2S) 2].* Journal of Materials Chemistry, 2001. **11**(5): p. 1486-1490.
